# Impact of environmental changes on the dynamics of temporal networks

**DOI:** 10.1371/journal.pone.0250612

**Published:** 2021-04-28

**Authors:** Hyewon Kim, Hang-Hyun Jo, Hawoong Jeong

**Affiliations:** 1 Asia Pacific Center for Theoretical Physics, Pohang, Republic of Korea; 2 Department of Physics, The Catholic University of Korea, Bucheon, Republic of Korea; 3 Department of Physics, Korea Advanced Institute of Science and Technology, Daejeon, Republic of Korea; 4 Center for Complex Systems, Korea Advanced Institute of Science and Technology, Daejeon, Republic of Korea; Universiteit Maastricht, NETHERLANDS

## Abstract

Dynamics of complex social systems has often been described in the framework of temporal networks, where links are considered to exist only at the moment of interaction between nodes. Such interaction patterns are not only driven by internal interaction mechanisms, but also affected by environmental changes. To investigate the impact of the environmental changes on the dynamics of temporal networks, we analyze several face-to-face interaction datasets using the multiscale entropy (MSE) method to find that the observed temporal correlations can be categorized according to the environmental similarity of datasets such as classes and break times in schools. By devising and studying a temporal network model considering a periodically changing environment as well as a preferential activation mechanism, we numerically show that our model could successfully reproduce various empirical results by the MSE method in terms of multiscale temporal correlations. Our results demonstrate that the environmental changes can play an important role in shaping the dynamics of temporal networks when the interactions between nodes are influenced by the environment of the systems.

## Introduction

Dynamical behaviors of various complex systems can be described by temporal patterns of interactions among constituents of the systems, which have recently been studied in the framework of temporal networks [1–3]. This is partly due to the increasing availability of datasets with high temporal resolution [4–8]. A number of temporal interaction patterns in natural and social phenomena are found to be non-Poissonian or bursty [7] and they have been known to strongly influence the dynamical processes taking place in the system, such as spreading and diffusion [9–18]. In addition, the dynamical properties of temporal networks were studied in terms of the effects of temporal resolution and time ordering of interactions [19–22]. To understand the underlying mechanisms behind empirical findings for temporal networks, several modeling approaches have been taken: These models could successfully generate characteristics of real-world temporal networks such as heavy-tailed degree distributions, community structure, and/or bursty behaviors [23–32], enabling us to better understand the interaction mechanisms in temporal networks.

In general, the dynamics of complex social systems is driven by both internal and external factors. The internal factors may correspond to the individual attributes or the nature of relationships between individuals. The internal factors may not be the only driving force for the bursty interaction patterns between individuals, which can also be affected by various external factors. The obvious external factors in human social behaviors are circadian, weekly, and even longer cycles, as evidenced by a number of empirical analyses [33–48]. Despite the importance of such external factors in understanding the temporal correlations observed in temporal networks, we find only few studies on the effects of external factors on bursty temporal interaction patterns. These effects have been studied, e.g., by modeling circadian and weekly patterns with a periodic event rate or activity level [33, 34] or by de-seasoning the cyclic behaviors from the bursty time series [35]. Our understanding of such effects is far from complete, which clearly calls for more rigorous and systematic studies.

In this paper, we investigate the impact of the time-varying external factors or environmental changes on temporal correlations in temporal networks. We first analyze the several temporal network datasets, some of which are known to be affected by the time-varying external factors, by means of the multiscale entropy (MSE) method [49, 50] for detecting temporal correlations in multiple timescales. This is because the time-varying external factors are expected to introduce non-trivial long-range temporal correlations in the dynamics of temporal networks. By the MSE method, we find that the datasets analyzed can be categorized according to the environmental similarity. Then we devise a temporal network model that considers both internal and external factors. Here the external factor is assumed to be periodic in time, while the internal one is constant of time. Incorporating the preferential interaction mechanism into the model, we successfully generate various patterns of temporal correlations in the temporal networks. Our modeling approach helps us better understand how the environmental changes may affect the non-trivial temporal interaction patterns observed in the empirical temporal networks.

## Related work

The effects of environmental changes on human interaction patterns have been investigated in a number of empirical analyses and by means of numerous model studies [33–48]. As each human action can be described by an event, the inter-event time distribution has been a relevant approach to the analysis. Thus, the effects of periodic external factors on bursty human dynamics have been studied in terms of heavy-tailed inter-event time distributions, in which each event can denote an individual action that is not necessarily an interaction with other individuals [34, 40] or an interaction between individuals [33–35, 37]. Such findings have been modeled, e.g., by inhomogeneous Poisson processes with time-varying event rates or activity levels [33, 34], time-dependent Hawkes processes with circadian cycles [51], and inhomogeneous Hawkes process with exogenous factors [52], as well as by using stochastic differential equations [43]. Note that most of the above studies focused on the analysis of a single time series of events even when those events indicate interactions between individuals. Thus, for more comprehensive approach to the bursty interaction dynamics, one can adopt the framework of temporal networks [1, 2] where the temporal network is typically defined as a set of interaction events between nodes in the network. To overcome the limitations of previous studies focused on the activity level of individuals, in our work, we study the influence of periodic external factors on the human dynamics at the system level by modeling temporal networks.

An activity-driven temporal network model might be one of the simplest models to present the highly dynamical interactions between nodes by using nodal activity [24]. The variants of this model have been used to generate the temporal and topological properties observed in several empirical datasets, such as heavy-tailed inter-event time distributions [28] and community structures [25, 30]. Most of the temporal network models have focused on the internal factors related to the individual attributes or the nature of relationships between individuals, such as nodal activity or memory effects between nodes. Therefore, it calls for modeling temporal networks with external factors and studying the external effects on the dynamics of temporal networks. We find only few studies on the effects of periodic external factors on the dynamics of temporal networks, e.g., see Ref. [48]. In our work, we introduce external factors into the activity-driven temporal network model to better understand the external effect in the dynamics of temporal networks.

We also find a variety of analysis methods to investigate the occurrence of changes in the dynamics of systems. Several methods have been developed to detect the changing point in topological changes, e.g., stochastic block model mechanisms [53, 54], and an information theoretical approach [55], and to measure temporal correlations considering high-order terms for finding the most relevant timescale on the dynamics of community structures or complex systems [56–58]. Other methods have considered the multiple timescales to detect short- and long-term temporal correlations [44, 49, 50]. In particular, the MSE method [49, 50], proposed to analyze temporal correlations across various timescales, is found to be a useful tool to identify differences in dynamical systems with multiple temporal correlations. Such a method can provide insight into the effect of environmental changes in complex systems because the timescales of the dynamics of environmental changes and human interactions can be different from each other. This method has been applied to datasets in a variety of fields, from biology to atmospheric science [49, 50, 59, 60], but as far as we know, it has not yet been applied to temporal network datasets. Here we apply this method to several face-to-face interaction datasets to analyze the temporal correlations of each dataset.

In this paper, we explore the effects of environmental changes in the dynamics of temporal networks through data analysis and network modeling. We consider the MSE method [49, 50] to analyze multiscale temporal correlations in several empirical temporal network datasets. Based on the empirical results, we propose a temporal network model to study the role of environmental changes in the dynamics of complex social systems. Our work provides quantified results of the impact of environmental changes on real-world complex systems in terms of multiple temporal correlations. It also contributes to a better understanding of the external effect in the dynamics of the temporal network by studying our model, which has been extended by introducing external factors, under various conditions.

## Methods

### Network-level time series of a temporal network

To characterize temporal correlations in temporal networks, we apply the multiscale entropy (MSE) method [49, 50] to the network-level time series of empirical face-to-face interaction datasets. As for the network-level time series, we first consider a time series for the number of interactions between individuals or activated links, as it is the simplest quantity measuring the overall interaction patterns of the temporal network. We also study the time series of the number of newly activated links or links that are activated for the first time. This quantity may capture the evolutionary dynamics of the network topology because the first activation of a link can be interpreted as the creation of the link.

**Fig 1:**
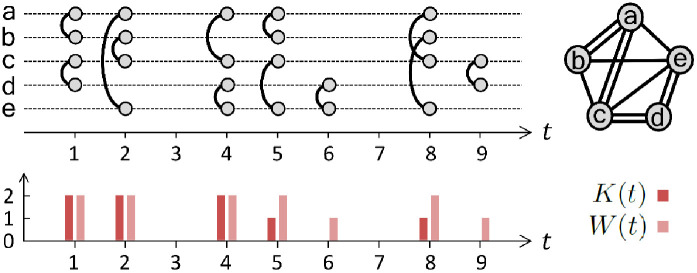
An example of a temporal network in discrete time. The time-resolved and time-aggregated representations of the network (top) and the network-level time series of {*K*(*t*)} and {*W*(*t*)} (bottom). Here *W*(*t*) and *K*(*t*) denote the number of activated links and the number of newly activated links at the time step *t*, respectively.

We introduce notations for the temporal network with *N* nodes, *K* links, and the total number of activations over all links *W* during the observation period of *T*. Each link *i* (*i* = 1, …, *K*) at a time step *t* (*t* = 1, …, *T*) can be either in an active state (interaction) or in an inactive state (no interaction), which are denoted by *A*_*i*_(*t*) = 1 and 0, respectively. The number of activated links at the time step *t*, denoted by *W*(*t*), is given as $W\left(t\right)=\sum_{i=1}^{K}A_{i}\left(t\right)$. Note that a weight of the link *i* can be obtained by $w_{i}\equiv \sum_{t=1}^{T}A_{i}\left(t\right)$. We also denote the number of newly activated links at the time step *t* by *K*(*t*). By definition *K*(*t*) ≤ *W*(*t*). The time series of *K*(*t*) and *W*(*t*) are written as {*K*(*t*)} and {*W*(*t*)}, respectively, where $\sum_{t=1}^{T}K\left(t\right)=K$ and $\sum_{t=1}^{T}W\left(t\right)=W$. Fig 1 shows an example of {*K*(*t*)} and {*W*(*t*)} for the temporal network with *N* = 5, *K* = 8, *W* = 12, and *T* = 9. The time-resolved and time-aggregated representations of the temporal network are shown in the top panels, while {*K*(*t*)} and {*W*(*t*)} are presented in the bottom panel.

Activity patterns in many temporal network datasets are known to be non-Poissonian or bursty [7], implying the existence of temporal correlations or memory effects, which are often found in multiple timescales. For characterizing the activity patterns with multiscale temporal correlations, we adopt the MSE method [49, 50]. It is because the face-to-face interaction datasets to be studied in our work have relatively short observation periods and the MSE method has been known to be less dependent on the time series length such as in physiological systems [49].

### Multiscale entropy

We present a brief review of the multiscale entropy (MSE) method [49, 50] for characterizing the time series with multiscale temporal correlations. Here, we describe the MSE method using the notations and symbols used in Refs. [49, 50].

We first define the sample entropy [61]. Let us consider a univariate discrete time series {*x*_*t*_} for *t* = 1, …, *T*, from which we get *T* − *m* vectors of length *m*, i.e., $X_{t}^{m}=\left(x_{t},\ldots ,x_{t+m-1}\right)$ for *t* = 1, …, *T* − *m*. For a given vector $X_{t}^{m}$, one can calculate the probability $C_{t}^{m}\left(r\right)$ that a random vector $X_{t^{\prime }}^{m}$ for *t*′ ≠ *t* lies within a distance *r* from $X_{t}^{m}$, namely, satisfying max{|*x*_*t*+*s*_ − *x*_*t*′+*s*_|}_*s* = 0, …, *m* − 1_ ≤ *r*. Then the average of $C_{t}^{m}\left(r\right)$ over *t* is denoted by $U^{m}\left(r\right)\equiv {\left(T-m\right)}^{-1}\sum_{t=1}^{T-m}C_{t}^{m}\left(r\right)$. Similarly, one can get *T* − *m* vectors of length *m* + 1, denoted by $X_{t}^{m+1}$, from which $C_{t}^{m+1}\left(r\right)$ and *U*^*m*+1^(*r*) are respectively calculated. Using *U*^*m*^(*r*) and *U*^*m*+1^(*r*) one defines the sample entropy, denoted by *S*_*E*_(*m*, *r*), as follows:

$$\begin{array}{c} S_{E}\left(m,r\right)\equiv -\ln\frac{U^{m+1}\left(r\right)}{U^{m}\left(r\right)}=\ln\frac{\sum_{t=1}^{T-m}C_{t}^{m}\left(r\right)}{\sum_{t=1}^{T-m}C_{t}^{m+1}\left(r\right)}. \end{array}$$ (1)

It is straightforward to see that the more random or complex time series tends to have the higher value of *S*_*E*_. However, the sample entropy cannot distinguish uncorrelated random time series from strongly correlated complex time series. To overcome this limit, Costa et al. [49] proposed the multiscale entropy method, which is to be discussed below.

**Fig 2:**
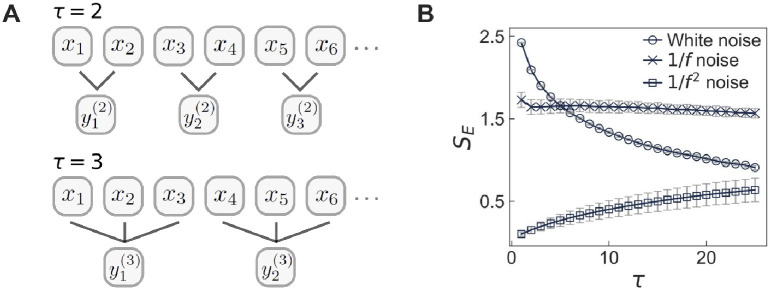
Multiscale entropy (MSE) method. (A) A schematic illustration of coarse-graining procedure of the time series {*x*_*t*_} for *t* = 1, …, *T* with the scale factor *τ* = 2 (top) and 3 (bottom). (B) Numerical results of the MSE method applied to the white noise, the 1/*f* noise, and the 1/*f*^2^ noise. Each value of *S*_*E*_ is averaged over 10 time series with *T* = 3 × 10^4^, and the error bar denotes its standard deviation.

To analyze the time series with multiscale temporal correlations by means of the sample entropy *S*_*E*_, Costa et al. proposed the MSE method [49] by incorporating a coarse-graining procedure. For a given time series {*x*_*t*_} for *t* = 1, …, *T* and the scale factor *τ*, the coarse-grained time series $\left\{y_{t}^{\left(\tau \right)}\right\}$ for *t* = 1, …, *T*/*τ* is constructed by averaging the elements in {*x*_*t*_} within non-overlapping time windows of size *τ* such that

$$\begin{array}{c} y_{t}^{\left(\tau \right)}=\frac{1}{\tau }\sum_{t^{\prime }=\left(t-1\right)\tau +1}^{t\tau }x_{t^{\prime }}\;\text{for}\;t=1,\cdots ,\frac{T}{\tau }, \end{array}$$ (2)

see also Fig 2(A). If *τ* = 1, the time series $\left\{y_{t}^{\left(1\right)}\right\}$ is the same as {*x*_*t*_}. The sample entropy *S*_*E*_ of $\left\{y_{t}^{\left(\tau \right)}\right\}$ is calculated for various values of *τ*, i.e., in various timescales, hence it is called the MSE method. This method has been applied to various datasets, e.g., for heartbeat, neural, and atmospheric time series [49, 50, 59, 60]. For the rest of the paper, we will calculate *S*_*E*_ in Eq (1) using *m* = 2 and *r* = 0.15*σ* with *σ* denoting the standard deviation of {*x*_*t*_} in all cases.

For the demonstration of the MSE method, we apply this method to three kinds of time series, i.e., white noise and 1/*f* noise as in Refs. [49, 50], and 1/*f*^2^ noise as in Ref. [62]. Here we generate a time series for white noise using random numbers that are drawn from a normal distribution with zero mean and variance of one. To generate a time series for 1/*f* noise we use the Voss-McCartney algorithm [63, 64]. Finally we generate a time series for 1/*f*^2^ noise using a Brown noise generator [65], which is essentially the temporal integration of white noise. The white noise is uncorrelated time series, while 1/*f* and 1/*f*^2^ noises are non-stationary as well as have strong temporal correlations with infinite memory and long-term memory in terms of decaying behaviors of autocorrelation functions, respectively [66, 67]. The results of the MSE method for these time series are presented in Fig 2(B). In the case of the white noise, *S*_*E*_ monotonically decreases as *τ* increases, whereas *S*_*E*_ remains almost constant for the 1/*f* noise, and *S*_*E*_ monotonically increases for the 1/*f*^2^ noise. The monotonically decreasing *S*_*E*_ for the white noise implies that the noisy, random behavior tends to be averaged out for the larger scale factor. In contrast, the overall constant *S*_*E*_ for the 1/*f* noise must be due to the temporal self-similarity of 1/*f* noise. Finally, the monotonically increasing *S*_*E*_ for the 1/*f*^2^ noise indicates that coarse-graining by the larger scale factor enhances the fluctuation of time series, hence makes the time series look more random.

## Empirical results for temporal networks

### Datasets

We consider six empirical face-to-face interaction datasets provided by the SocioPatterns project [68]: a primary school dataset for 2 days, a hospital dataset for 5 days from 6 a.m. to 8 p.m. for each day, a workplace dataset for 10 days, a high school dataset for 4 days in 2011, a high school dataset for 7 days in 2012, and a conference dataset for 3 days. The high school datasets in 2011 and 2012 are denoted as “school (2011)” and “school (2012)”, respectively. Table 1 provides information about the datasets, including the observation period in terms of the number of days and the number of distinct nodes in each dataset. In all datasets, contacts or interactions between individuals were recorded every 20 seconds, defining the unit of the time step in our work.

**Table 1:** Information about the datasets. or each dataset, we present the observation period in terms of the number of days, the minimum and maximum numbers of nodes in the daily partition of the dataset (*N*_min_ and *N*_max_), and the number of distinct nodes in the entire dataset (*N*_tot_), respectively.

Dataset	Primary school	Hospital	Workplace	School (2011)	School (2012)	Conference
days	2	5	10	4	7	3
*N*_min_	236	40	59	112	145	97
*N*_max_	238	50	72	121	158	102
*N*_tot_	242	75	92	126	180	113

### Topological properties of time-aggregated networks

**Fig 3:**
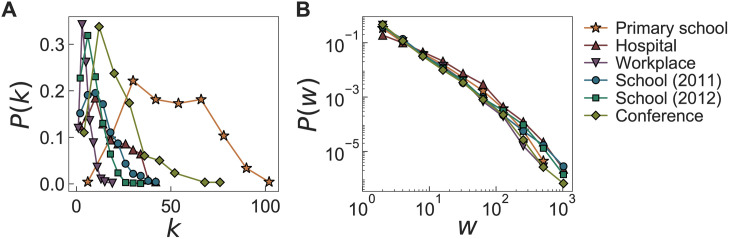
Degree distributions *P*(*k*) and weight distributions *P*(*w*) for empirical datasets. (A) *P*(*k*) and (B) *P*(*w*) of time-aggregated networks for six face-to-face interaction datasets, i.e., for the primary school (✰), hospital (△), workplace (▽), school (2011) (○), school (2012) (□), and conference (◊). *P*(*k*)s are linearly binned, while *P*(*w*)s are logarithmically binned.

We first investigate the basic topological properties of time-aggregated networks. We obtain the time-aggregated network for each day of each dataset to get degree and weight distributions, where the degree *k* means the number of neighbors. These daily distributions are averaged for each dataset to get the averaged *P*(*k*) and *P*(*w*), as shown in Fig 3. We observe that *P*(*k*)s show increasing and then decreasing behaviors, while being mostly right-skewed, except for the case with the primary school. *P*(*k*) for the primary school shows both large average and large variance of degrees. The weight distributions *P*(*w*) are found to show the similar heavy-tailed behaviors across all datasets. We conclude that the topological structures of time-aggregated networks of six datasets are qualitatively similar to each other.

### Multiscale entropy analysis on empirical datasets

**Fig 4:**
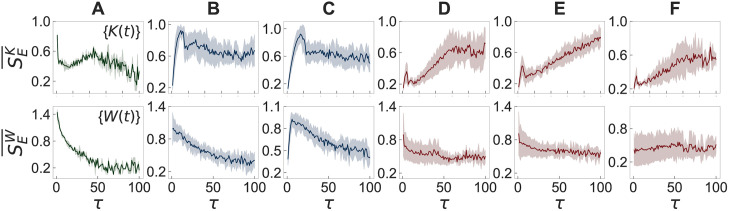
Results of the MSE method for empirical datasets. Results of the MSE method applied to time series {*K*(*t*)} (top panels) and {*W*(*t*)} (bottom panels), respectively denoted as $\bar{S_{E}^{K}}$ and $\bar{S_{E}^{W}}$, of six face-to-face interaction datasets: (A) primary school, (B) hospital, (C) workplace, (D) school (2011), (E) school (2012), and (F) conference. See the main text for details.

Next, we apply the MSE method to the time series {*K*(*t*)} and {*W*(*t*)} derived from the above mentioned datasets. For each day of each dataset, we calculate the sample entropy *S*_*E*_ for the coarse-grained time series using the scale factor of *τ* = 1, …, 100. Then the curves of *S*_*E*_ as a function of *τ* are averaged over all days for each dataset, denoted by $\bar{S_{E}}$. The results of $\bar{S_{E}}$ are presented with the corresponding standard deviations in Fig 4, where the top (bottom) panels show the results for {*K*(*t*)} ({*W*(*t*)}). From now on we denote $\bar{S_{E}}$ for {*K*(*t*)} and {*W*(*t*)} as $\bar{S_{E}^{K}}$ and $\bar{S_{E}^{W}}$, respectively.

According to the behavioral patterns of $\bar{S_{E}^{K}}$, the six datasets can be divided into three categories: (i) The primary school dataset shows the overall increasing and then decreasing behavior of $\bar{S_{E}^{K}}$, apart from the peak at *τ* = 1 [top panel in Fig 4(A)]. Note that the decreasing behavior of $\bar{S_{E}^{K}}$ was observed for the white noise in Fig 2(B). (ii) $\bar{S_{E}^{K}}$ for hospital and workplace datasets increases quickly and then decreases very slowly or even fluctuates around some constant [top panels in Fig 4(B) and 4(C)]. The behavior that remains almost constant is similar to the result for the 1/*f* noise in Fig 2(B), implying the long-range temporal correlations. (iii) The other three datasets, i.e., school (2011), school (2012), and conference, show the overall increasing $\bar{S_{E}^{K}}$ [top panels in Fig 4(D)–4(F)], indicating that the time series appears to be more complex when looked at in longer timescales. Note that the increasing behavior of $\bar{S_{E}^{K}}$ was observed for the 1/*f*^2^ noise in Fig 2(B), and also the similar increasing behaviors have been reported for neural time series [69–71].

It is remarkable to mention that in all cases we find a peak around at a small value of *τ* whether the peak is a global maximum (primary school, hospital, and workplace) or a local maximum (school (2011), school (2012), and conference). These peaks may indicate that all time series we analyzed contain random noise of short timescales to some extent, which is however effectively averaged out in the procedure of coarse-graining with larger values of *τ*.

Results for {*W*(*t*)} in the bottom panels of Fig 4 can be better understood by comparing them with those for {*K*(*t*)} as *W*(*t*) is the sum of *K*(*t*) and the number of activated links that have been activated before the time *t*. The latter kind of activations, corresponding to *W*(*t*) − *K*(*t*), indeed leads to different behaviors of $\bar{S_{E}^{W}}$ than $\bar{S_{E}^{K}}$: In the case with the primary school, $\bar{S_{E}^{W}}$ overall monotonically decreases, implying that the values of {*W*(*t*)} are more uncorrelated with each other than those of {*K*(*t*)}. $\bar{S_{E}^{W}}$ for the hospital and workplace datasets overall decreases for the almost entire range of *τ*, implying the long-range correlations in {*K*(*t*)} must have been largely destroyed in {*W*(*t*)}. Finally, the other three datasets for school (2011), school (2012), and conference show the almost flat behaviors of $\bar{S_{E}^{W}}$, similarly to the case with 1/*f* noise. In sum, we find that the activations observed by *W*(*t*) − *K*(*t*) tend to weaken the temporal correlations present in {*K*(*t*)}.

### Heterogeneity level and memory coefficient

For more detailed understanding of the empirical results by the MSE method, we introduce two quantities for characterizing {*K*(*t*)} and {*W*(*t*)}: the heterogeneity level *H* and the memory coefficient *M*. These quantities are based on the burstiness parameter and memory coefficient that were originally proposed in Ref. [72] for measuring the temporal correlations in the point processes in terms of interevent times. In our work, instead of interevent times, we analyze the values of time series of {*x*_*t*_} for *t* = 1, …, *T*. To measure how broad the distribution of values of *x*_*t*_ is compared to their mean, we calculate the mean and standard deviation of the values of *x*_*t*_, respectively denoted by *m*_*x*_ and *σ*_*x*_, to define the heterogeneity level *H* as follows:

$$\begin{array}{c} H\equiv \frac{\sigma_{x}-m_{x}}{\sigma_{x}+m_{x}}. \end{array}$$ (3)

If all values of *x*(*t*) are the same, one gets *H* = −1, while *H* = 0 in the case when *x*_*t*_ is exponentially distributed. If the distribution of *x*_*t*_ is heavy tailed, *H* > 0 is expected. The memory coefficient *M* for the time series of {*x*_*t*_} is defined as

$$\begin{array}{c} M\equiv \frac{1}{T-1}\sum_{t=1}^{T-1}\frac{\left(x_{t}-m_{1}\right)\left(x_{t+1}-m_{2}\right)}{\sigma_{1}\sigma_{2}}, \end{array}$$ (4)

where *m*_1_ and *σ*_1_ (*m*_2_ and *σ*_2_) are the mean and standard deviation of {*x*_1_, …, *x*_*T*−1_} ({*x*_2_, …, *x*_*T*_}), respectively. The value of *M* ranges from −1 to 1. If a large (small) *x*_*t*_ tends to be followed by the large (small) *x*_*t*+1_, *M* is positive, while *M* is negative in the opposite case.

**Fig 5:**
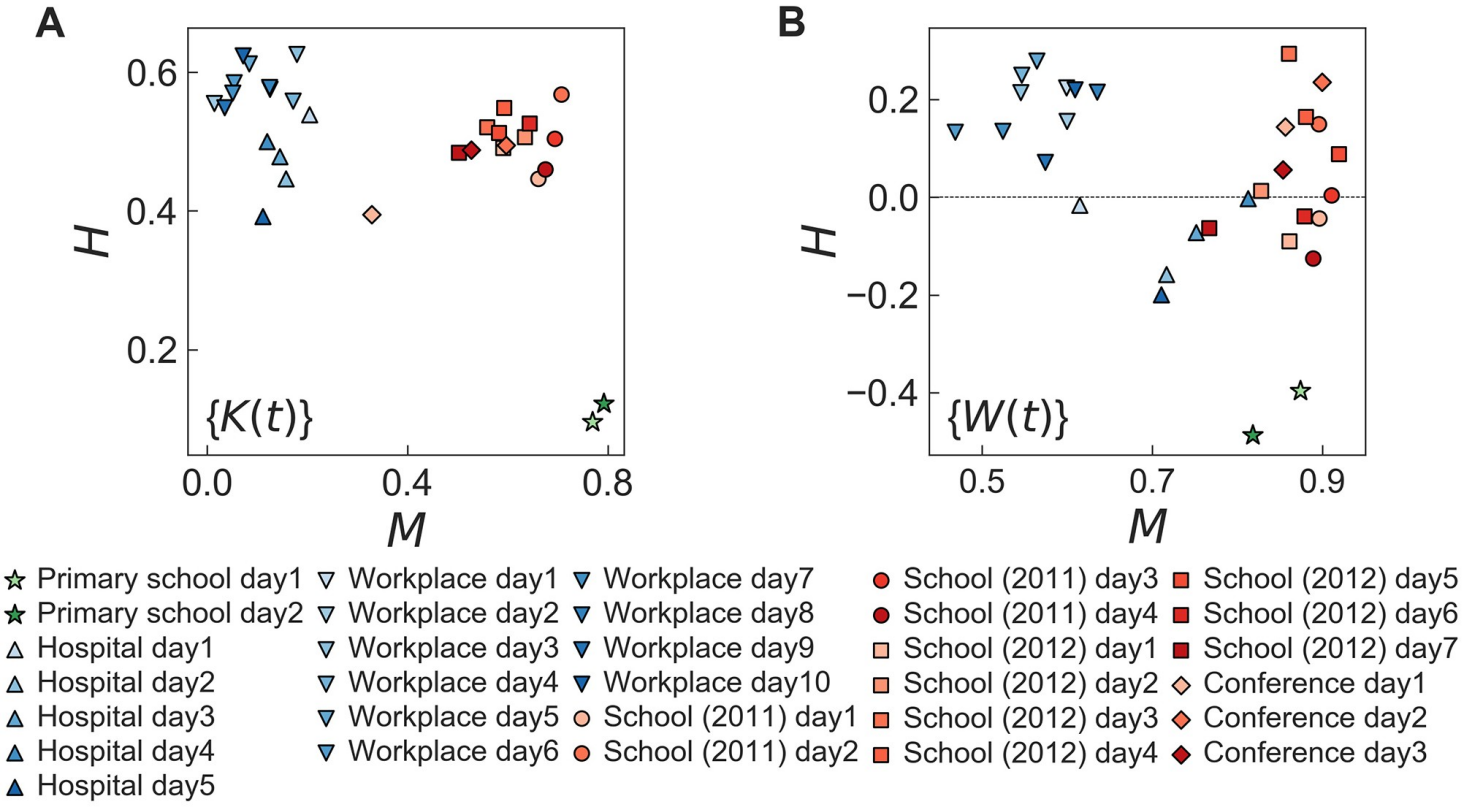
Scatter plot of values of *H* and *M* for empirical datasets. *H* in Eq (3) and *M* in Eq (4) in the (*M*, *H*)-space for time series (A) {*K*(*t*)} and (B) {*W*(*t*)} are presented using the same datasets analyzed in Fig 4.

We calculate the values of *H* and *M* for {*K*(*t*)} and {*W*(*t*)} for each day of each dataset. These values are plotted in the (*M*, *H*)-spaces as shown in Fig 5. In the case with {*K*(*t*)}, we clearly find three clusters of points: (i) The primary school dataset is characterized by the smallest values of *H* (≈0.1) and the largest values of *M* (≈0.8), implying that the values of the time series are relatively homogeneous, while they are strongly correlated with each other. (ii) The hospital and workplace datasets show the large values of *H* (0.4 ≲ *H* ≲ 0.7) and the small values of *M* (0 ≲ *M* ≲ 0.2). It means that the values of the time series are highly heterogeneous, but showing with relatively weak correlations between them. (iii) The other three datasets, i.e., school (2011), school (2012), and conference, show large values of both *H* and *M* such that the values of the time series are highly heterogeneous as well as strongly correlated with each other. From the results for {*W*(*t*)}, we can observe three clusters similarly to those for {*K*(*t*)}, apart from the observation that the values of *H* (*M*) are overall much smaller (larger) than those of {*K*(*t*)}.

We remark that three clusters identified in the (*M*, *H*)-spaces one-to-one correspond to three different behavioral patterns of *S*_*E*_ as a function of *τ* as discussed above. From such a correspondence one can guess that heterogeneous values of the time series, i.e., large *H*, are necessary to show the non-decreasing behaviors of *S*_*E*_. Further, the increasing *S*_*E*_ could additionally require strong positive correlations between consecutive values of the time series.

Interestingly, the datasets in each cluster turn out to share similar social conditions either enhancing or suppressing interactions between individuals. In particular, we focus on the temporal behaviors of such conditions or environmental changes. The participants in the primary school dataset could have break times but only three times including lunch per day [5], while in the high school and conference cases, the interaction between participants were affected by scheduled programs with several breaks [73, 74]. During the breaks participants have chances to introduce each other or strengthen their existing relations, while such interactions can be relatively suppressed for the rest of the observation periods. Unlike schools and conference, there were no constrained schedules for the participants in the hospital and workplace datasets [75, 76]. Generally speaking, the environmental changes can obviously influence the evolution of temporal networks, yet the effects of environmental changes on the evolution of temporal networks are far from being fully understood. To explore such effects, in the following Section we will devise and study a temporal network model that qualitatively reproduces the observed patterns by incorporating the environmental changes.

## Temporal network model

To explore the impact of environmental changes on the dynamics of temporal networks, we will first investigate a model for generating a periodic time series by considering both external and internal factors. This time series could represent either {*K*(*t*)} or {*L*(*t*)}, where *L*(*t*)≡*W*(*t*) − *K*(*t*) denotes the number of links that have previously been activated and are activated at the time step *t* as well. Then, based on the periodic time series model, we will devise and study a temporal network model showing various temporal interaction patterns observed in the empirical datasets.

### Modeling a periodic time series

We devise a model for generating a periodic time series {*z*(*t*)} for *t* = 1, …, *T*, whose values are determined by both the external and internal factors. Considering the fact that the value of the time series of our interest is not always positive in the empirical analysis, we introduce the probability of having a positive *z*(*t*), which is denoted by *ρ* (0 < *ρ* < 1). Then one can write

$$\begin{array}{c} z\left(t\right)=\left\{ \begin{array}{ll} 0 & \text{with}\;1-\rho \text{,}\\ l & \text{with}\;\rho , \end{array}\right. \end{array}$$ (5)

where the positive integer *l* is drawn from an exponential distribution *P*(*l*;λ(*t*)) with a time-varying parameter λ(*t*), that is,

$$\begin{array}{c} P\left(l;\lambda \left(t\right)\right)=\lambda {\left(t\right)}^{-1}e^{-l/\lambda (t)}. \end{array}$$ (6)

The time-varying parameter λ(*t*) can be written as λ_int_(*t*) + λ_ext_(*t*), where λ_int_(*t*) and λ_ext_(*t*) are the rates of spontaneous and externally-driven activations, respectively. We assume that λ_int_(*t*) is constant of time, i.e., λ_int_(*t*) = λ_int_, while λ_ext_(*t*) is a periodic function whose time average vanishes. The positive (negative) λ_ext_(*t*) enhances (suppresses) activations compared to the constant activity level of λ_int_.

**Fig 6:**
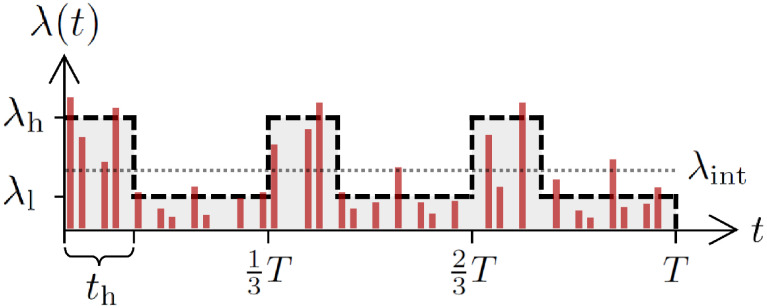
An illustration of the model for generating a periodic time series. The periodic time series {*z*(*t*)} for *t* = 1, …, *T* (red vertical lines) is generated using Eqs (5) and (6), where the time-varying parameter λ(*t*) in Eq (6) (thick dashed curve) is shaped by three parameters, i.e., λ_h_, λ_l_, and *t*_h_, in the case with *n* = 3. The horizontal dotted line for λ_int_ is plotted for comparison. See the main text for details.

For simplicity, we assume that λ(*t*) has only two levels of activity, i.e., λ_h_ and λ_l_ (λ_h_ ≥ λ_l_). To be precise, the total period *T* is divided into *n* intervals. Each interval of length *T*/*n* starts with a high activity period of length *t*_h_, for which λ(*t*) = λ_h_. This is followed by a low activity period of length *T*/*n* − *t*_h_, for which λ(*t*) = λ_l_. Note that *t*_h_ ≤ *T*/*n*. In Fig 6 we present an example of λ(*t*) for the case with *n* = 3. The sum of *z*(*t*) over the entire period of *T* is assumed to be given as a control parameter *Z*, namely,

$$\begin{array}{c} Z=\sum_{t=1}^{T}z\left(t\right)=\rho \sum_{t=1}^{T}\lambda \left(t\right)=\rho [\lambda_{h}t_{h}+\lambda_{l}(\frac{T}{n}-t_{h})]n, \end{array}$$ (7)

leaving us with two independent parameters out of λ_h_, λ_l_, and *t*_h_, provided that *Z*, *T*, *ρ*, and *n* are fixed. The external effect can be controlled mainly by the ratio λ_h_/λ_l_ and *t*_h_, where the larger ratio tends to be associated with the shorter period of *t*_h_. The case with λ_h_/λ_l_ = 1 implies no external effect (λ_ext_(*t*) = 0), leading to λ(*t*) = λ_int_ = *Z*/(*ρT*), which is also obtained when *t*_h_ = *T*/*n*.

**Fig 7:**
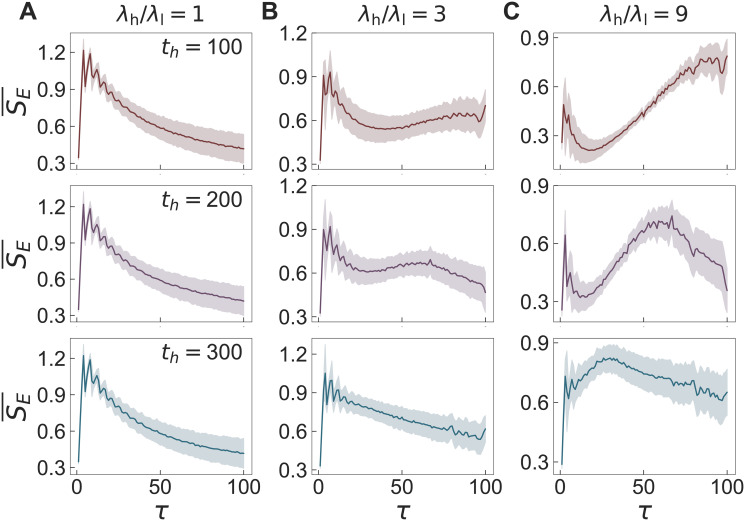
Results of the MSE method for the periodic time series model. Results of the MSE method applied to the time series {*z*(*t*)} using the periodic time series model with *Z* = 1000, *T* = 2000, *ρ* = 0.2, and *n* = 5 for values of λ_h_/λ_l_ = 1, 3, and 9 (left to right) and *t*_h_, 200, and 300 (top to bottom). For each panel, we have generated 10^3^ time series to get the averaged curve $\bar{S_{E}}$ as a function of the scale factor *τ*.

For each combination of λ_h_/λ_l_ and *t*_h_, we generate 10^3^ time series {*z*(*t*)} with fixed values of *Z* = 1000, *T* = 2000, *ρ* = 0.2, and *n* = 5. The multiscale entropy (MSE) method is applied to each time series to get the averaged curve of $\bar{S_{E}}$ as a function of the scale factor *τ*, as shown in Fig 7. In the case without external effect, i.e., λ_h_/λ_l_ = 1, we observe the overall decreasing behavior of $\bar{S_{E}}$, which was observed in the white noise [Fig 2(B)]. As expected, *t*_h_ has no effects on the results. As the periodic external effect gets stronger with the larger values of λ_h_/λ_l_, we find overall flat or even increasing behaviors of $\bar{S_{E}}$, as depicted in Fig 7(B) and 7(C). Note that the overall flat behavior of $\bar{S_{E}}$ for λ_h_/λ_l_ = 3 and *t*_h_ = 100 was observed in the analysis of 1/*f* noise [Fig 2(B)]. Furthermore, it turns out that as *t*_h_ increases from 100, the range of *τ* for the flat or increasing $\bar{S_{E}}$ shrinks and it is followed by the decreasing $\bar{S_{E}}$ for the large *τ* regime.

For understanding the effects of *t*_h_ on the MSE results in the general case with λ_h_/λ_l_ > 1, we calculate the fluctuation of λ(*t*) as follows:

$$\begin{array}{c} \sigma_{\lambda }^{2}\equiv \frac{1}{T}\sum_{t=1}^{T}{\left[\lambda \left(t\right)-\lambda_{\text{int}}\right]}^{2}=\frac{\lambda_{\text{int}}^{2}{\left(\lambda_{h}/\lambda_{l}-1\right)}^{2}s\left(1-s\right)}{{\left[1+\left(\lambda_{h}/\lambda_{l}-1\right)s\right]}^{2}}, \end{array}$$ (8)

where *s* ≡ *t*_h_/(*T*/*n*). The fluctuation $\sigma_{\lambda }^{2}$ is found to be a decreasing function of *t*_h_ in the range of *t*_h_ ≥ *T*/[*n*(λ_h_/λ_l_ + 1)]. For our case with λ_h_/λ_l_ = 3 and *T*/*n* = 400, the fluctuation $\sigma_{\lambda }^{2}$ decreases for *t*_h_ ≥100, implying that λ(*t*) approaches the constant function, i.e., λ(*t*) = λ*int*. Hence the overall decreasing behavior of $\bar{S_{E}}$ is expected for large values of *t*_h_.

Our results for the periodic time series model enable us to get insight into the empirical findings, i.e., decreasing, flat, and/or increasing $\bar{S_{E}}$, from the temporal network datasets in the previous Section to a large extent.

### Modeling temporal networks

Using the periodic time series model in the previous Subsection, we now devise a temporal network model that generates various temporal interaction patterns by considering both external and internal factors. We assume that the periodically changing environment affects not only the topological structure of the network, i.e., newly activated links, but also the activity patterns of links that have previously been activated. The topological structure of the network evolves according to the activity-driven network model [24–30], where each node is activated at its given activity rate to make connections to other nodes. For the activity patterns of previously activated links, we incorporate a preferential activation mechanism for the heavy-tailed weight distributions in Fig 3(B), which is inspired by a preferential attachment mechanism accounting for the power-law degree distributions in scale-free networks [77].

We introduce our temporal network model as follows: At the time step *t* = 0, we consider a network of *N* isolated nodes in which each node *i* is assigned an activity *a*_*i*_ that is drawn from an activity distribution *F*(*a*). At each time step *t* (*t* = 1, …, *T*), the number of newly activated links *K*(*t*) and the number of activations for previously activated links *L*(*t*) are given by assuming that both *K*(*t*) and *L*(*t*) are affected by the periodically changing environment in a similar way. Therefore, the same periodic time series model in the previous Subsection can be used for both *K*(*t*) and *L*(*t*) but with different parameter values. Precisely, we use the symbols *ρ*_*K*_, λ_*K*,h_, and λ_*K*,l_ (*ρ*_*L*_, λ_*L*,h_, and λ_*L*,l_) for modeling *K*(*t*) (*L*(*t*)), while *T*, *n*, and *t*_h_ have the same values for *K*(*t*) and *L*(*t*). These parameters should satisfy the following relations:

$$\begin{array}{c} K=\sum_{t=1}^{T}K\left(t\right)=\rho_{K}[\lambda_{K,h}t_{h}+\lambda_{K,l}(\frac{T}{n}-t_{h})]n, \end{array}$$ (9)

$$\begin{array}{c} L=\sum_{t=1}^{T}L\left(t\right)=\rho_{L}[\lambda_{L,h}t_{h}+\lambda_{L,l}(\frac{T}{n}-t_{h})]n. \end{array}$$ (10)

Then *K*(*t*) nodes are randomly chosen with probabilities proportional to their activities (*a*_*i*_). For each chosen node *i*, we randomly choose another node *j* that has never been connected to the node *i* up to the time step *t* − 1. The link between nodes *i* and *j* is created and activated.

For the activation of previously activated links, we first denote by *E*_*t*_ the set of links that have previously been activated up to the time step *t* − 1. Note that $|E_{t}|=\sum_{t^{\prime }=1}^{t-1}K\left(t^{\prime }\right)$. Then *L*(*t*) links are randomly chosen from *E*_*t*_ according to the preferential activation mechanism to be activated. By the preferential activation mechanism the more active links in the past are more likely to be activated in the future, which is expected to result in the heavy-tailed weight distributions in the time-aggregated networks. The *L*(*t*) links are chosen with probabilities proportional to their accumulated weights up to the time step *t* − 1, i.e.,

$$\begin{array}{c} \Pi_{i}\left(t\right)=\frac{w_{i}\left(t-1\right)}{\sum_{j\in E_{t}}w_{j}\left(t-1\right)}, \end{array}$$ (11)

where $w_{i}\left(t\right)\equiv \sum_{t^{\prime }=1}^{t}A_{i}\left(t^{\prime }\right)$. In the early stage of the simulation, *L*(*t*) may exceed |*E*_*t*_|, in which case a new random number is drawn from the distribution in a form of Eq (6) until *L*(*t*) ≤ |*E*_*t*_| is satisfied. Finally, *W*(*t*) is given as *K*(*t*) + *L*(*t*) at each time step *t*.

Every activation at the time step *t* lasts only for one time step before the next time step *t* + 1 begins. The sum of *K*(*t*) and *L*(*t*) over the entire period of *T* is denoted by *K* and *L*, respectively, defining the total number of activations across all links *W* ≡ *K* + *L*.

### Role of external effect in temporal networks

We generate temporal networks using our temporal network model. Based on the empirical degree distributions in Fig 3(A), all nodes are considered to have the same activity, i.e., *a*_*i*_ = *a* for *i* ∈ {1, …, *N*}. We perform the simulations with the fixed values of *N* = 100, *K* = 1000, *W* = 10000, *T* = 2000, *ρ*_*K*_ = 0.2, *ρ*_*L*_ = 0.8, and *n* = 5, but for various combinations of *t*_h_, λ_*K*,h_/λ_*K*,l_, and λ_*L*,h_/λ_*L*,l_. Here the fixed values of parameters are based on the statistics of related quantities derived from datasets except for *n*. As the choice of *n* is not obvious from some datasets, we have referred to either the number of breaks (in some datasets) or the number of peaks in the time series for the number of interactions (in other datasets). We consider three cases with parameter values of (*t*_h_, λ_*K*,h_/λ_*K*,l_, λ_*L*,h_/λ_*L*,l_) = (200, 3, 1) (“Case 1”), (100, 3, 1) (“Case 2”), and (100, 9, 1.5) (“Case 3”). These cases correspond to three categories identified by the empirical analysis of six face-to-face datasets in the previous Section: Case 1 is for the primary school dataset, Case 2 is for the hospital and workplace datasets, and Case 3 is for the school (2011), school (2012), and conference datasets. For each case, 10^3^ temporal networks are generated for analysis.

**Fig 8:**
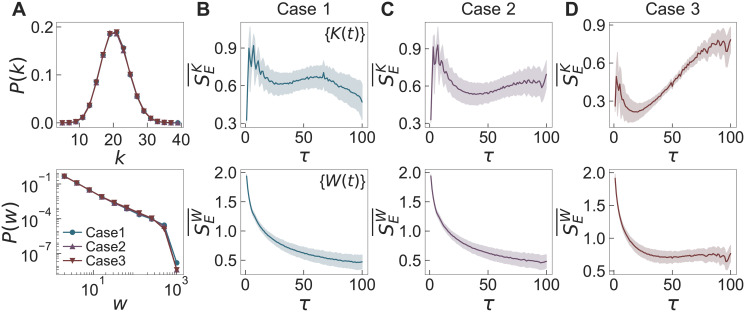
Simulation results of the temporal network model. (A) Degree and weight distributions of time-aggregated networks and (B–D) the results by the MSE method applied to {*K*(*t*)} (top panels) and {*W*(*t*)} (bottom panels), denoted as $\bar{S_{E}^{K}}$ and $\bar{S_{E}^{W}}$, respectively. For each combination of parameter values (*t*_h_, λ_*K*,h_/λ_*K*,l_, λ_*L*,h_/λ_*L*,l_) = (200, 3, 1) (“Case 1”), (100, 3, 1) (“Case 2”), and (100, 9, 1.5) (“Case 3”), we generate 10^3^ temporal networks using the fixed values of *N* = 100, *K* = 1000, *W* = 10000, *T* = 2000, *ρ*_*K*_ = 0.2, *ρ*_*L*_ = 0.8, and *n* = 5. In panel (A), degree distributions *P*(*k*) are linearly binned, while weight distributions *P*(*w*) are logarithmically binned.

From the generated temporal networks, we first measure the degree and weight distributions of the time-aggregated networks, as shown in Fig 8(A). For all cases, *P*(*k*)s are binomial distributions as expected from the assumption that *a*_*i*_ = *a* for all nodes *i*, and *P*(*w*)s show heavy tails due to the preferential activation mechanism. Then, by applying the MSE method to the time series of {*K*(*t*)} and {*W*(*t*)}, we calculate $\bar{S_{E}^{K}}$ and $\bar{S_{E}^{W}}$ with their standard deviations, as shown in Fig 8(B)–8(D). It turns out that our temporal network model successfully generates various temporal interaction patterns observed in the empirical datasets using the above parameter values of (*t*_h_, λ_*K*,h_/λ_*K*,l_, λ_*L*,h_/λ_*L*,l_). For example, in Fig 8(D), $\bar{S_{E}^{K}}$ ($\bar{S_{E}^{W}}$) shows overall increasing (flat) behaviors, which have been observed in the analysis of datasets for school (2011), school (2012), and conference [see Fig 4(D)–4(F)].

**Fig 9:**
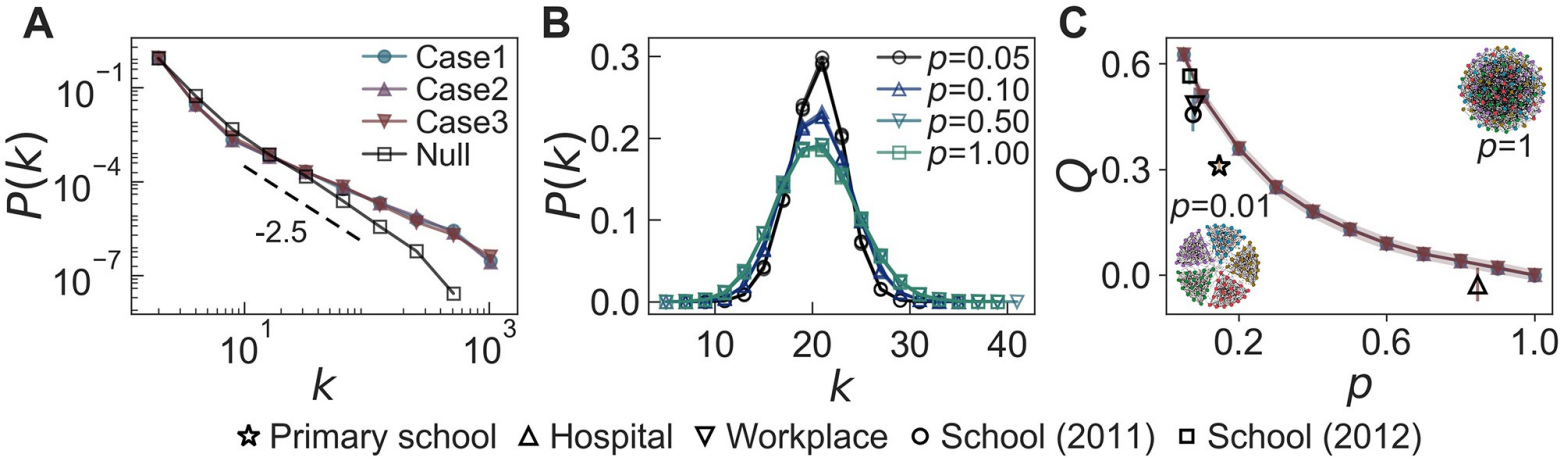
Simulation results of the temporal network model considering heterogeneous activities of nodes or the community property. (A) The networks are generated with *F*(*a*) ∼ *a*^−2.5^ and *N* = 10^4^, and the other parameters are the same as in Fig 8. (B) and (C) The networks are generated with *N*_*c*_ = 5 in the same parameter settings as in Fig 8. In the panels (A) and (B), degree distributions *P*(*k*)s are logarithmically and linearly binned, respectively. In the panel (C), the modularity *Q* is shown for various *p*. Each empty marker presents the empirical result for the primary school (✰), hospital (△), workplace (▽), school (2011) (○), and school (2012) (□).

Although we do not observe broad degree distributions in the empirical datasets analyzed, we test the effect of heterogeneous activities on the network structure. In many real-world networks, the activities of nodes are known to be heterogeneous [1, 2, 4, 6–9]. We generate temporal networks using *F*(*a*) ∼ *a*^−*γ*^ with *γ* = 2.5 for networks of size *N* = 10^4^, whereas other parameters have the same values as in Fig 8. In Fig 9(A), we find that *P*(*k*)s of the time-aggregated networks in all cases are essentially the same, also showing heavier tails than *F*(*a*). For comparison, we calculate the *P*(*k*) in the case when every node has the chance to get activated at each time step as in the original model [24]: As expected, we find the same power-law exponent in *P*(*k*) as *γ* in *F*(*a*) [see the “Null” case in Fig 9(A)]. Thus, heavier tails of *P*(*k*)s in our model might be due to the limited activations of nodes by *K*(*t*), effectively enhancing the activations of more active nodes than less active ones.

In addition, we also study the effect of community structure, such as classes in school, by considering *N*_*c*_ non-overlapping communities with *N*/*N*_*c*_ nodes in each community. Each of *K*(*t*) activated nodes at the time step *t* may create a link to another random node that belongs to the same community or another community. We define a parameter *p* as the ratio of the probability that the activated node chooses a neighbor node from one of other communities to the probability that the activated node chooses a neighbor node belonging to the same community. If *p* = 0, no links between nodes in different communities are created, while when *p* = 1, the activated node randomly chooses its neighbor node from the entire network, leading to the network without community structure.

We generate temporal networks for *N*_*c*_ = 5 with the same parameter values as in Fig 8, also assuming that *a*_*i*_ = *a* for all nodes *i*. Fig 9(B) and 9(C) respectively show the degree distribution *P*(*k*) and modularity *Q* [78] of the time-aggregated networks for various *p*. When *p* = 1, we obtain *Q* ≈ 0, which corresponds to the results in Fig 8. As *p* decreases, the activated nodes tend to form links with nodes belonging to the same community. As a result, *Q* increases, while the variance of *P*(*k*) decreases. The results show that even if the external factors have the same effect on the number of activated links, the topological structure of the network can be different depending on the connectivity patterns of nodes.

The empirical datasets we analyze provide information on the communities to which the nodes belong, except for the conference one: 11 communities in the primary school, 4 communities in the hospital, 5 communities in the workplace, 4 communities in the school (2011), and 5 communities in the school (2012). We estimate the value of *p* by counting the number of links within the same communities. In Fig 9(C), we plot *Q* of the empirical datasets as a function of *p*, which is presented by empty markers with the corresponding standard deviations. Each marker denotes an averaged value for the daily datasets. We can confirm that the hospital and workplace datasets have network structures with a significant difference in terms of *Q*, although they show similar MSE results, as shown in Fig 4(B) and 9(C). This emphasizes the observation that even if the environmental impacts on networks are similar, the topological properties can vary depending on the connectivity patterns.

By the numerical simulation of our temporal network model, we have shown how the periodic external factor, when combined with the internal factor and the preferential activation mechanism, can induce complex temporal correlations in the network-level interaction patterns over a wide range of timescales.

Finally, we remark that in our model the links are created (or activated for the first time) in different times, which may introduce some aging effects to the dynamics of temporal networks, e.g., as discussed in Ref. [28]. The different creation times of links can also affect the dynamical processes taking place in temporal networks such as spreading [79, 80]. In this sense our results highlight the need to study the impact of the environmental changes on the dynamics of temporal networks.

## Conclusion

The impact of environmental changes on the dynamics of temporal networks has been widely recognized, yet its understanding is far from complete. In our work we have analyzed six face-to-face interaction datasets in the framework of temporal networks by applying the multiscale entropy (MSE) method to the network-level time series. Based on the MSE results, we find that the temporal interaction patterns in those datasets can be categorized according to the environmental similarity, such as similar patterns of classes or break times in schools. To investigate the effects of periodic external factors on the various temporal interaction patterns, we first devise a model for generating a periodic time series to show that our model can reproduce various behaviors of the MSE results. Then we devise a temporal network model, based on the periodic time series model, that successfully generates various temporal interaction patterns in temporal networks. We also incorporate the preferential activation mechanism to account for the heavy-tailed distributions of link weights.

Our results demonstrate the importance of the environmental factors in understanding the dynamics of temporal networks. In particular, one can further investigate the possibilities of classifying the datasets according to the environmental similarity by applying our analysis method to other temporal network datasets. In addition, we have studied a temporal network model mainly focusing on the periodic external factors with only two levels of activity and on the relatively simple networks, while our model can be extended to take into account more realistic features such as complex social relations between individuals and more realistic cyclic behaviors of environmental changes.
